# Optimize the dose of oxaliplatin for locally advanced rectal cancer treated with neoadjuvant chemoradiotherapy followed by radical surgery and adjuvant chemotherapy

**DOI:** 10.1186/s12885-020-06988-x

**Published:** 2020-06-01

**Authors:** Hui Chang, Ya-lan Tao, Wu Jiang, Chen Chen, Shi-liang Liu, Wei-jun Ye, Yuan-hong Gao

**Affiliations:** 1 Department of Radiation Oncology, Sun Yat-sen University Cancer Center, Guangzhou, China; 2 State Key Laboratory of Oncology in South China, Collaborative Innovation Center for Cancer Medicine, Guangzhou, China; 3 Department of Colorectal Surgery, Sun Yat-sen University Cancer Center, Guangzhou, China

**Keywords:** Rectal neoplasms, Oxaliplatin, Distant metastasis, Survival

## Abstract

**Background:**

Addition of oxaliplatin to capecitabine remains controversial for locally advanced rectal cancer (LARC). And cumulative oxaliplatin dose (COD) varied among clinical trials showing different therapeutic effects of this regimen. The objective of this study was to explore how COD affected tumor metastasis and patient survival.

**Methods:**

Totally 388 patients diagnosed with stage cII-III rectal cancer and treated with neoadjuvant chemoradiotherapy followed by radical surgery plus adjuvant chemotherapy were consecutively enrolled into this study and retrospectively reviewed. After grouping by total chemotherapy cycle (TCC), influences of COD on adverse effects and patients’ survivals were analyzed in each group. Univariate and multivariate survival analyses were performed through Kaplan-Meier approach and COX proportional hazards model, respectively. Age, gender, anemia, differentiation, carcinoembryonic antigen, carbohydrate antigen 19–9, pretreatment clinical stage and postsurgical pathologic stage were used as covariates.

**Results:**

COD < 460 mg/m^2^ emerged as an independent predictor of poorer overall, metastasis-free and disease-free survivals, in patients treated with TCC ≤ 7. The hazard ratios were 1.972, 1.763 and 1.637 (*P* values were 0.021, 0.028 and 0.041), respectively. But it was note-worthy that COD ≥460 mg/m^2^ increased incidence of acute toxicities from 38.4 to 70.8% (*P* < 0.001). And in patients treated with TCC ≥ 8, COD failed to be a prognosticator.

**Conclusions:**

For LARC patients treated with insufficient TCC (≤ 7), oxaliplatin of ≥460 mg/m^2^ might be needed to improve survival, though it might resulted in more acute toxicities.

## Background

Rectal cancer accounts for 56.7% of colorectal cancer, which is the 3rd most common malignancies in China [1, 2]. Currently, the standard mode to manage locally advanced rectal cancer (LARC) is neoadjuvant chemoradiotherapy (NACRT) followed by radical surgery and adjuvant chemotherapy (ACT) [3]. In spite of ideal local control, distant metastasis (DM) will finally occur in 20.6% of LARC patients [4]. Namely, DM is the primary style of treatment failure in these patients. It is essential to reduce DM, so as to improve prognosis of LARC.

Chemotherapy is one of the most effective treatments to eliminate DM of solid tumors. For LARC, capecitabine plus oxaliplatin (CAPEOX) is one of the commonly used chemotherapy regimens. Yet, clinical values of oxaliplatin still remain controversial [5]. Several phase 3 trials showed that neither improved local control nor survival benefit was attained through adding oxaliplatin to fluorouracil or capecitabine [6–8]. By contrast, different results were seen in the recent CAO/ARO/AIO-04 trial. Oxaliplatin led to a 4% elevation of both pathological complete response (pCR) rate and 3-year disease-free survival (DFS). When chemotherapy intensity was taken into account, the CAO/ARO/AIO-04 trial had an obviously higher cumulative oxaliplatin dose (COD) than prior studies (1000 vs. 250–360 mg/m^2^) [9]. So we speculated that treatment effects of oxaliplatin might depend on COD.

On the other side, oxaliplatin could lead to a series of toxicities, especially acute and late peripheral neuropathy (PN). PN is a dose-limit toxicity because it lacks efficacious therapeutic methods. And it is reported that the incidence of PN increases dramatically when COD exceeded 780 mg/m^2^, which is often used as an upper limit of COD [10]. Here we conducted a study to optimize COD for LARC patients, within the tolerable dose range. Since the National Comprehensive Cancer Network recommended the total cycle of CAPEOX chemotherapy be ≥8, which was also confirmed in our previous works to bring better overall survival (OS), DFS and metastasis-free survival (MFS) [11], analyses of survivals and toxicities were made in patients with total chemotherapy cycle (TCC) ≤ 7 and ≥ 8, respectively.

## Methods

### Patients

A patient would be enrolled and retrospectively reviewed, if he or she had: (i) age ≤ 75 years old; (ii) rectal cancer initially diagnosed in our hospital between Jan. 1st 2007 and Mar. 31st 2014; (iii) pretreatment stage of cII-III (cT3-4N0M0, cT1-4 N1-2 M0); (iv) completed records of NACRT and radical (R0) resection; (v) COD ≤780 mg/m^2^. COD was defined as the total dose of oxaliplatin received by a patient in neoadjuvant chemotherapy (NACT) and ACT. The exclusion criteria included: (i) performance score of the Eastern Cooperative Oncology Group > 2; (ii) severe heart, lung, hepatic, kidney or hematopoietic dysfunctions unfit for chemotherapy or radiotherapy; (iii) past history of other cancerous diseases, chemotherapy or radiotherapy; (iv) DM before or during radiotherapy; (v) application of monoclonal antibody.

### Diagnostic work-up

The pathological diagnosis was made through rectoscope. To decide pretreatment clinical stage, computed tomography (CT) of chest and abdomen, magnetic resonance imaging (MRI) of pelvis and endoscopic ultrasonography was performed for each patient. Whole-body bone scan was performed routinely to exclude bone metastasis. Positron emission tomography would be performed when suspicious DM was discovered by CT or MRI. Serum levels of carcinoembryonic antigen (CEA) and carbohydrate antigen 19–9 (CA19–9) were also tested before treatment.

### Treatment strategies

Radiotherapy was performed with a 3-dimensional conformal or intensity-modulated technique, using a linear accelerator delivering an 8-MV photon beam. Target delineation was done on basis of the guidelines of the International Commission on Radiation Units and Measurements Reports 50 and 62. Total doses of 50 and 46 Gy were prescribed to macroscopic tumor (containing primary lesion and enlarged lymph nodes) and high-risk (containing pararectal, presacral, obturator, internal and common iliac) lymphatic drainage regions, respectively. Irradiation was performed in a conventional fractionation (2 Gy per fraction, 1 fraction per day, 5 days per week).

CAPEOX was used as the regimen of both NACT and ACT. The dose of capecitabine and oxaliplatin were 1000 mg/m^2^ twice daily on Days 1–14 and 130 mg/m^2^ on Day 1, repeated every 21 days. NACT was administered in 2 modes: (i) totally 2 cycles during radiotherapy; (ii) totally 4 cycles, including 1, 2 and 1 cycles before, during and after radiotherapy, respectively. During radiotherapy, the dose of oxaliplatin was reduced to 100 mg/m^2^. After surgery, 4 cycles of ACT was performed. In ACT, single-agent capecitabine would take place of CAPEOX if: (i) COD reached 780 mg/m^2^; (ii) grade 3/4 PN of the Common Terminology Criteria for Adverse Events (CTCAE) happened; (iv) grade 4 thrombocytopenia of CTCAE happened twice.

Radical surgery was scheduled 6–8 weeks after the last cycle of NACT, according to the standard of total mesorectal excision (TME). It contained complete removal of tumor-located rectal segment (with a 2-cm margin), mesorectum (with a 5-cm distal margin) and surrounding lymphovascular fatty tissue. A precise, sharp dissection was made within presacral space to ensure completeness of pelvic visceral fascia. If an adjacent organ was infiltrated by tumor, partial or total of it would be removed. When completeness of TME is doubted, a frozen section of resection margin was subjected to intraoperative pathologic examination. TME quality and resected specimens were examined by two pathologists specialized in gastrointestinal cancers. Macroscopically visible tumor block was embedded in paraffin and go through serial 3- to 5-mm full thickness sections. Pathologic evaluation contained infiltration and differentiation of tumor, numbers of examined and involved lymph nodes, and tumor regression grade (TRG).

Staging in this study was based on the TNM staging classification (8th edition) of the Union for International Cancer Control / American Joint Cancer Committee, both before treatment and after surgery. TME quality was assessed based on MECURY criteria [12]. TRG was based on Mandard’s five-tier grading system [13], in which TRG 1–2 and 3–5 were defined as good and bad responders, respectively. Chemoradiotherapy-related acute toxicities were assessed based on the CTCAE criteria version 4.03.

### Follow-up

In the first 3 years after treatment, the patients were followed up every 3–6 months through outpatient interview. Complete physical examination, thoraco-abdominal CT, pelvic MRI, serum CEA and CA19–9 evaluation were performed at every interview. Rectoscope and whole-body bone scan were performed annually. After the 3rd year, the patients were followed up every 6–12 months through outpatient interview or telephone, until death from rectal cancer (confirmed by death certificates) or Dec. 31st 2018, whichever came first.

### Endpoint definition

OS was used as the primary endpoint in this study. It was defined as the percentage of patients who still survived after a defined duration from pathologic diagnosis. The secondary endpoints included DFS, recurrence-free survival (RFS) and MFS. These 3 endpoints were the percentages of patients without corresponding events after a defined duration, also from pathologic diagnosis. The events for RFS and MFS were local recurrence (LR) and DM, respectively. Death, LR and DM were all considered as the events for DFS. The patients were regarded censored if they had no event of death, LR or DM, or were lost to follow-up until Dec. 31st 2018. The percentages of patients with acute toxicities, PN and grade 3/4 acute toxicities were also used as secondary endpoints. Acute toxicities referred to toxicities happening in the period of NACRT and ACT.

### Statistical analysis

The eligible patients were divided by TCC, into the TCC ≤ 7 and ≥ 8 groups. First, comparability and adverse effects between the patients receiving different COD were assessed in each group, through a Chi-square test. Next, predictive ability of COD on patients’ survival was analyzed through a Kaplan-Meier approach-based univariate analysis. Last, a multivariate analysis based on COX proportional hazards model was made to confirm predictive independence of COD. Hazard ratios (HR) and 95% confidence interval (CI) were calculated for each factor during survival analysis.

The candidate prognosticators in univariate analysis included age, gender (male vs. female), anemia (yes vs. no), poor differentiation (yes vs. no), CEA, CA19–9, pretreatment clinical stage (cIII vs. cII), postsurgical pathologic stage (ypIII-II vs. ypI-0), TRG (5–3 vs. 2–1) and COD. A factor would enter multivariate analysis when it exhibited statistical significance in univariate analysis. The cutoff value of age was the median age of the whole cohort. The definition of anemia was hemoglobin < 130 g/L for male and < 120 g/L for female, in accordance with the standard of the World Health Organization [14]. The upper normal limit of serum CEA and CA19–9 were defined according to the standard of our hospital (5.0 ng/ml and 35 U/ml, respectively) [11]. The median CODs of the TCC ≤ 7 and ≥ 8 groups were used as their cutoff values of COD, respectively.

**Fig. 1:**
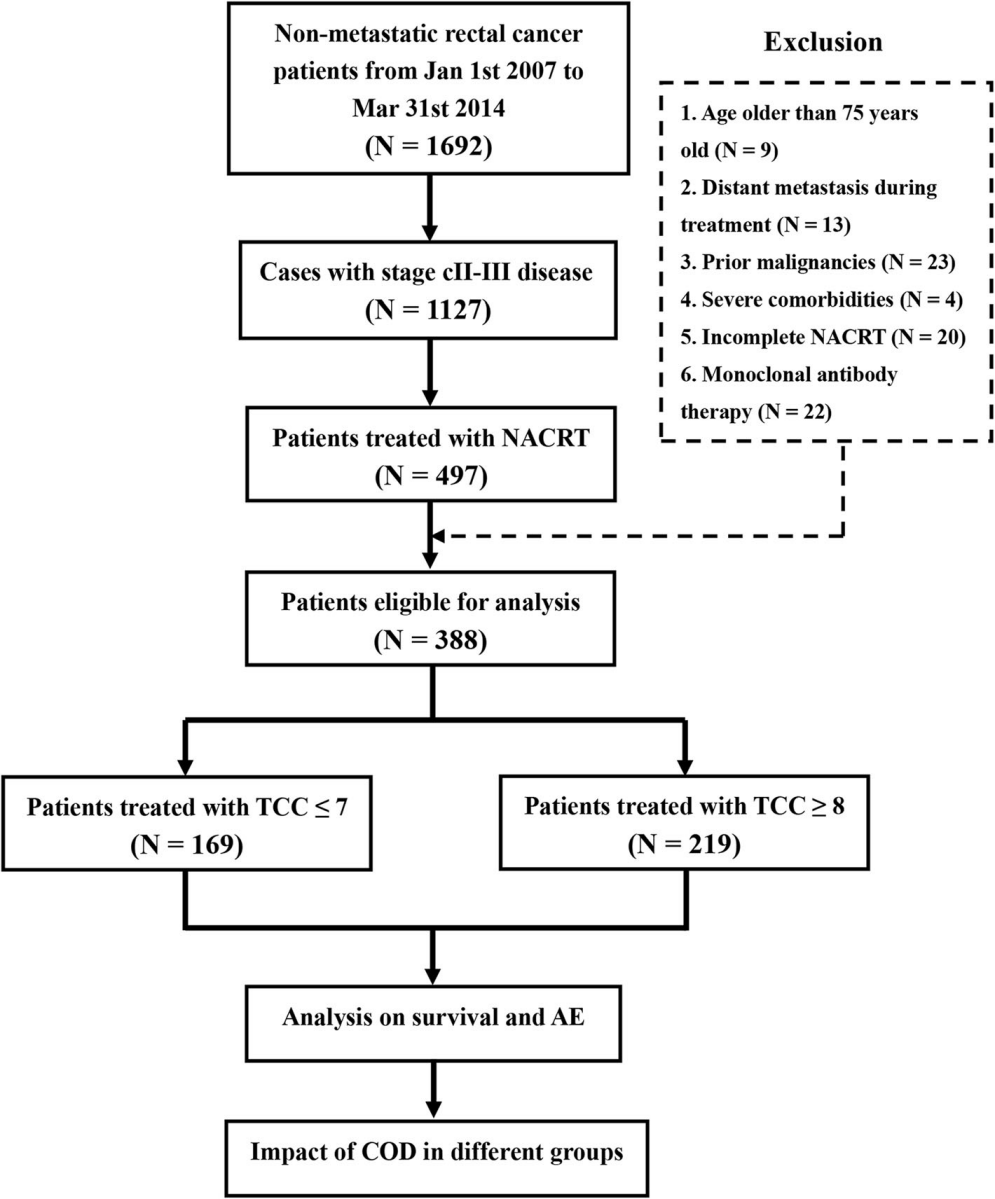
Procedure of enrollment and analysis. Abbrevations: NACRT, neoadjuvant chemoradiotherapy; TCC, total chemotherapy cycle; COD, cumulative oxaliplatin dose; AE, adverse effect

Statistical analysis was done by IBM SPSS Statistics 23.0 (IBM Co., Armonk, New York, US**)**. A difference with a two-sided *P* value of < 0.05 was considered to be statistically significant. The whole procedure of this study was summarized as Fig. 1.

## Results

### Patient enrollment

From January 2007 to March 2014, there were totally 1127 consecutive patients diagnosed with untreated LARC in our hospital. Among these patients, 479 cases received NACRT followed by radical surgery. And finally 388 patients were eligible for analysis, after exclusion of 9 cases with age > 75 years old, 13 cases with DM during treatment, 23 cases with prior malignancies, 4 cases with severe comorbidities, 20 cases with incomplete NACRT, and 22 cases receiving monoclonal antibody therapy. Among the 388 patients, 378 (97.4%) and 10 (2.6%) cases underwent complete (MECURY I) and nearly complete (MECURY II) mesorectal excision. There was no incomplete (MECURY III) mesorectal excision.

### Baseline characteristics

The median age of the patients was 55 (range, 15 to 75) years old. Thus, the cutoff value of age was 55 years old. When grouped by TCC, there were 169 and 219 patients in the TCC ≤ 7 and ≥ 8 groups, respectively. In the TCC ≥ 8 group, 3 (1.4%) cases received 10 cycles of chemotherapy (6 cycles of CAPEOX and 4 cycles of capecitabine). The other 216 (98.6%) cases all received 8 cycles. In the TCC ≤ 7 group, 111 (65.9%), 45 (26.6%) and 13 (7.7%) cases received 6, 4 and 2 cycles, respectively. In these 2 group, the median CODs were 460 (range, 200–720) and 720 (range, 200–780) mg/m^2^, which were also used as cutoff values of COD for subsequent analysis.

The baseline clinical features of the patients were showed in Table 1, including age, gender, anemia, tumor differentiation, CEA, CA19–9 and clinical stage. No difference was observed between the patients receiving different COD, in either the TCC ≤ 7 or ≥ 8 group.

**Table 1:** Clinical characteristics of the eligible patients

Characteristics	TCC ≤ 7 (***N*** = 169)			TCC ≥ 8 (***N*** = 219)		
	COD < 460 mg/m^**2**^ (***N*** = 73)	COD ≥ 460 mg/m^**2**^ (***N*** = 96)	***P*** value	COD < 720 mg/m^**2**^ (***N*** = 90)	COD ≥ 720 mg/m^**2**^ (***N*** = 129)	***P*** value
**Age / years old**						
**≥ 55**	44 (60.3%)	55 (57.3%)	0.697	45 (50.0%)	51 (39.5%)	0.125
**< 55**	29 (39.7%)	41 (42.7%)		45 (50.0%)	78 (60.5%)	
**Gender**						
**Male**	46 (63.0%)	71 (74.0%)	0.127	63 (70.0%)	78 (60.5%)	0.147
**Female**	27 (37.0%)	25 (26.0%)		27 (30.0%)	51 (39.5%)	
**Anemia**						
**Yes**	26 (35.6%)	27 (28.1%)	0.298	33 (36.7%)	35 (27.1%)	0.134
**No**	47 (64.4%)	69 (71.9%)		57 (63.3%)	94 (72.9%)	
**Differentiation**						
**Poor**	11 (16.7%)	12 (12.9%)	0.506	8 (8.9%)	20 (15.5%)	0.149
**Moderate-good**	62 (83.3%)	84 (87.1%)		82 (91.1%)	109 (84.5%)	
**CEA / ng/ml**						
**≥ 5**	36 (49.3%)	39 (46.0%)	0.260	42 (46.7%)	56 (43.4%)	0.634
**< 5**	37 (50.7%)	57 (54.0%)		48 (53.3%)	73 (56.6%)	
**CA19–9 / U/ml**						
**≥ 35**	18 (24.7%)	15 (15.6%)	0.581	11 (12.2%)	23 (17.8%)	0.260
**< 35**	55 (75.3%)	81 (84.4%)		79 (87.8%)	106 (82.2%)	
**Clinical stage**						
**cIII**	47 (64.4%)	73 (76.0%)	0.098	71 (78.9%)	112 (86.8%)	0.119
**cII**	26 (35.6%)	23 (24.0%)		19 (21.1%)	17 (13.2%)	

*Abbreviations*: *TCC* Total chemotherapy cycle, *COD* Cumulative oxaliplatin dose, *CEA* Carcinoembryonic antigen, *CA19–9* Carbohydrate antigen 19–9

### Adverse events

**Fig. 2:**
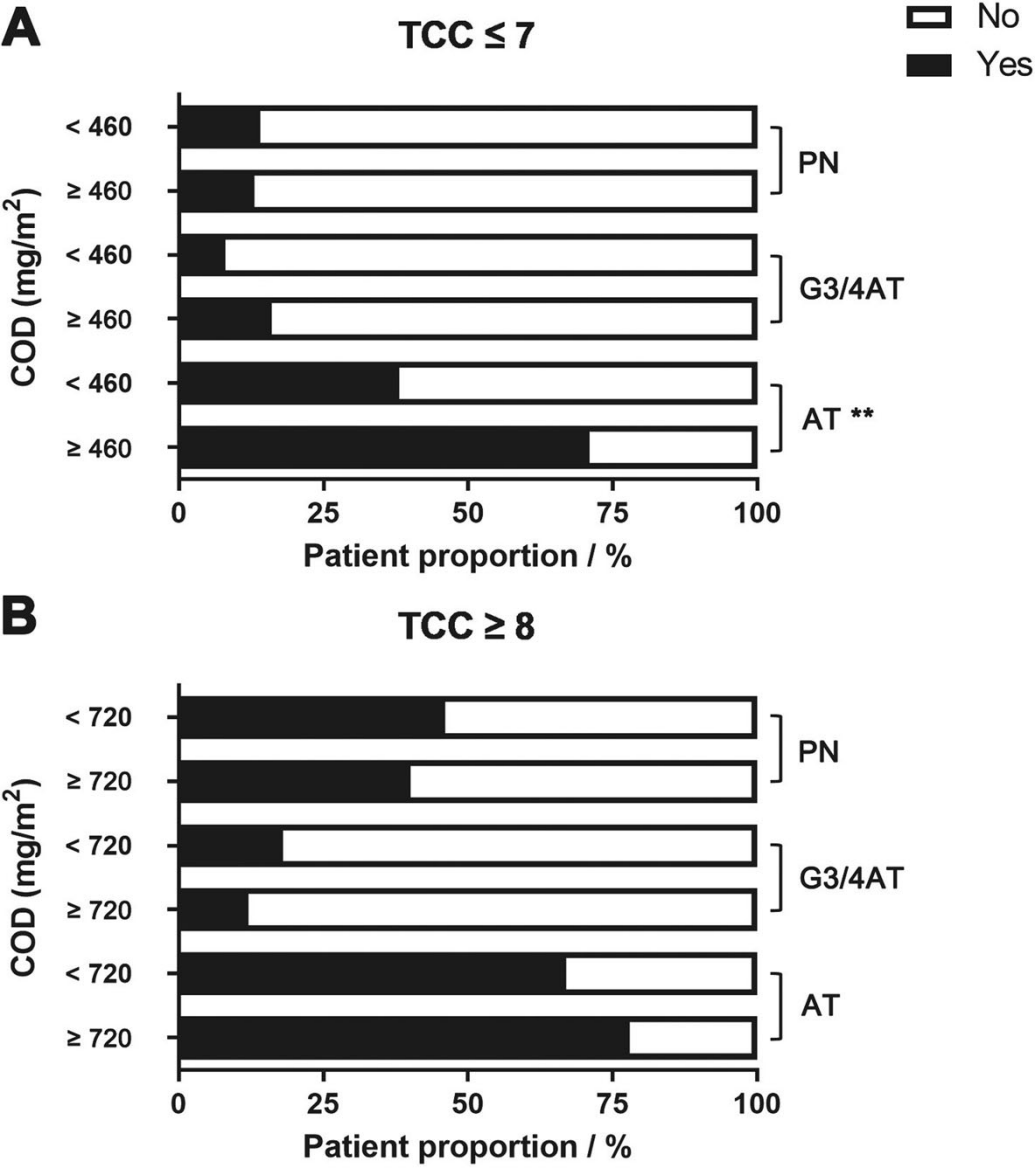
Adverse events in patients treated with different cumulative oxaliplatin doses. **a:** total chemotherapy cycle ≤7**. b:** total chemotherapy cycle ≥8**.** Abbreviations: COD, cumulative oxaliplatin dose; TCC, total chemotherapy cycle; AT, acute toxicities; G3/4AT, grade 3/4 acute toxicities; PN, peripheral neuropathy. ** *P* < 0.01

In the TCC ≤ 7 group, the patients treated with COD ≥460 mg/m^2^ had a higher incidence of acute toxicities, compared with those treated with COD < 460 mg/m^2^ (70.8% vs. 38.4%, *P* < 0.001). However, there was no difference in incidence of PN or grade 3/4 acute toxicities between patients receiving different CODs (Fig. 2a).

In the TCC ≥ 8 group, no difference was seen in incidence of acute toxicities, PN or grade 3/4 acute toxicities, between the patients receiving COD ≥720 mg/m^2^ and those treated with COD < 720 mg/m^2^ (Fig. 2b).

### Survival outcomes

**Fig. 3:**
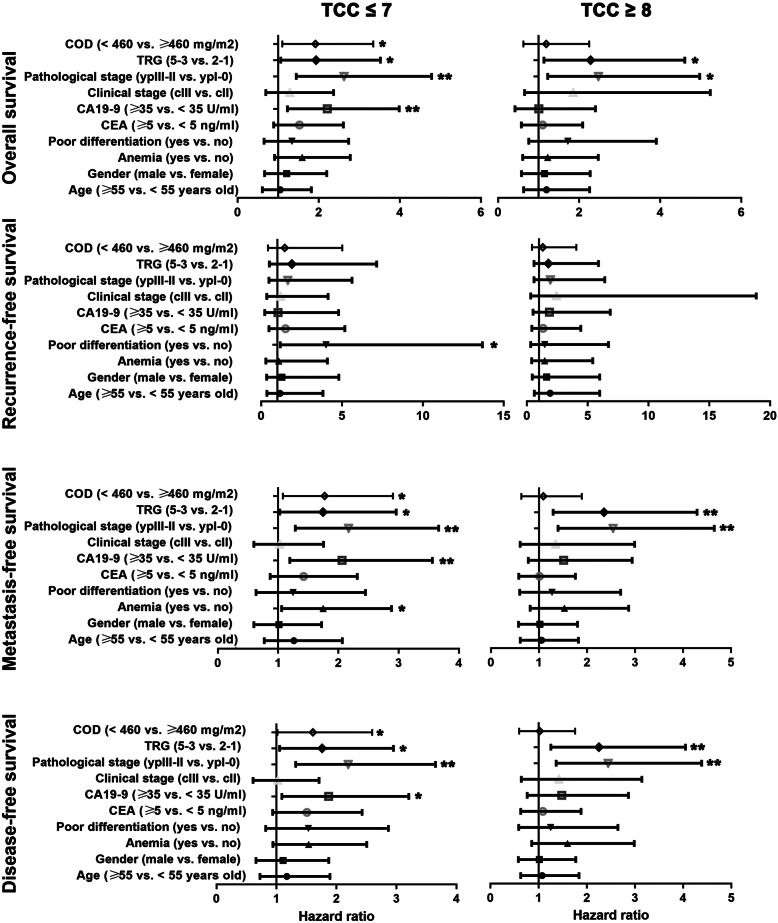
Univariate survival analysis in patients treated with different total chemotherapy cycles. Abbreviations: TCC, total chemotherapy cycle; COD, cumulative oxaliplatin dose; TRG, tumor regression grade; CEA, carcinoembryonic antigen; CA19–9, carbohydrate antigen 19–9. * *P* < 0.05, ** *P* < 0.01

Results of univariate analysis were showed in Fig. 3. In the TCC ≤ 7 group, CA19–9 ≥ 35 U/ml, stage ypIII-II disease, TRG 5–3 and COD < 460 mg/m^2^ were risk factors of poorer OS (*P* values were 0.008, 0.002, 0.031 and 0.021), MFS (*P* values were 0.009, 0.004, 0.039 and 0.024) and DFS (*P* values were 0.023, 0.002, 0.031 and 0.037). Poor differentiation was the sole risk factor of poorer RFS (*P* = 0.027). And anemia was also a risk factor of poorer MFS (*P* = 0.029).

In the TCC ≥ 8 group, stage ypIII-II disease and TRG 5–3 were risk factors of poorer OS (*P* values were 0.011 and 0.021), MFS (*P* values were 0.002 and 0.005) and DFS (*P* values were 0.003 and 0.006). COD (< 720 vs. ≥ 720 mg/m^2^) failed to be a predictor of OS, RFS, MFS or DFS.

**Fig. 4:**
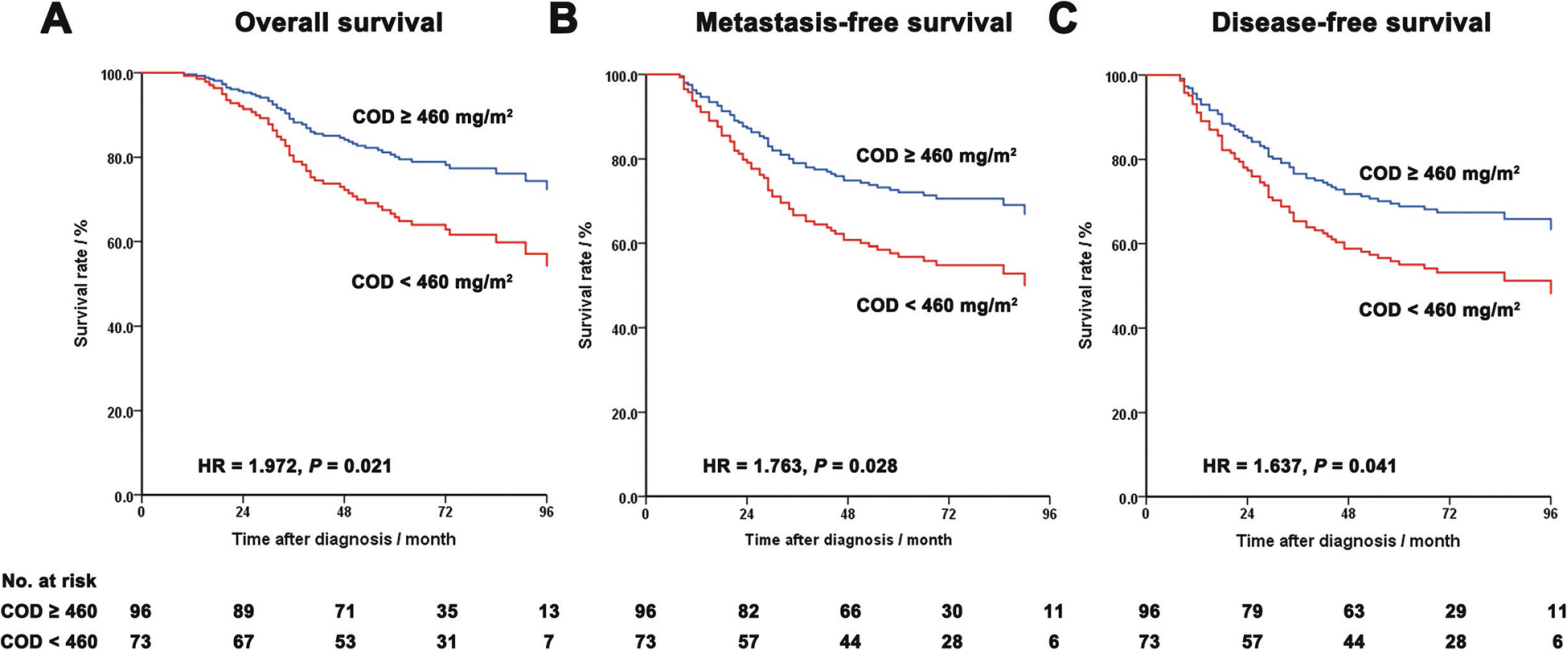
COX regression-based survival curves of patients treated with total chemotherapy cycle ≤7. **a**: overall survival. **b**: metastasis-free survival **c**: disease-free survival. Abbreviations: COD, cumulative oxaliplatin dose; HR, hazard ratio; CI, confidence interval

In multivariate analysis on the TCC ≤ 7 group (Table 2), postsurgical pathologic stage and COD maintained as independent predictors of OS, MFS and DFS. The HRs of postsurgical pathologic stage (ypIII-II vs. ypI-0) were 4.237 (95% CI, 1.252–14.29), 2.747 (95% CI, 1.068–7.812) and 2.801 (95% CI, 1.053–7.462), respectively. And the HRs of COD (< 460 vs. ≥ 460 mg/m^2^) were 1.972 (95% CI, 1.106–3.521), 1.763 (95% CI, 1.062–2.933) and 1.637 (95% CI, 1.110–2.688), respectively. Survival curves of patients divided by COD were showed in Fig. 4. Both postsurgical pathologic stage and TRG failed to be an independent predictor of OS, MFS or DFS, in the TCC ≥ 8 group (See supplementary materials, Table S1).

**Table 2:** Multivariate survival analysis in patients treated with total chemotherapy cycle ≤7

**Factors of OS**	***P*value**	**HR**	**95% CI**
Pathological stage (ypIII-II vs. ypI-0)	0.020*	4.237	1.252–14.29
COD (< 460 vs. ≥ 460 mg/m^2^)	0.021*	1.972	1.106–3.521
CA19–9 (≥ 35 vs. < 35 U/ml)	0.247	1.448	0.774–2.709
TRG (5–3 vs. 2–1)	0.316	1.842	0.558–6.077
**Factors of MFS**	***P*value**	**HR**	**95% CI**
Pathological stage (ypIII-II vs. ypI-0)	0.048*	2.747	1.068–7.812
COD (< 460 vs. ≥ 460 mg/m^2^)	0.028*	1.763	1.062–2.933
CA19–9 (≥ 35 vs. < 35 U/ml)	0.161	1.503	0.850–2.656
Anemia (yes vs. no)	0.114	1.514	0.906–2.530
TRG (5–3 vs. 2–1)	0.427	1.530	0.536–4.369
**Factors of DFS**	***P*value**	**HR**	**95% CI**
Pathological stage (ypIII-II vs. ypI-0)	0.039*	2.801	1.053–7.462
COD (< 460 vs. ≥ 460 mg/m^2^)	0.041*	1.637	1.110–2.688
CA19–9 (≥ 35 vs. < 35 U/ml)	0.264	1.381	0.783–2.438
TRG (5–3 vs. 2–1)	0.499	1.398	0.529–3.690

* *P* < 0.05. *Abbreviations*: *OS* Overall survival, *MFS* Metastasis-free survival, *DFS* Disease-free survival, *HR* Hazard ratio, *CI* Confidence interval, *COD* Cumulative oxaliplatin dose, *CA19–9* Carbohydrate antigen 19–9, *TRG* Tumor regression grade

## Discussion

Fluorouracil, or its derivative capecitabine, is now the basis of chemotherapy for LARC. Combining fluorouracil or capecitabine to preoperative radiotherapy has been proven to improve local control, due to radiosensitizing effects of these 2 agents. However, no improved OS is brought by concomitant chemoradiotherapy [15, 16]. Oxaliplatin is another radiosensitizing agent that emerges to be more effective than fluorouracil [17]. And it has been demonstrated by clinical studies to improve DFS, and even OS of colon cancer, when added to fluorouracil-based chemotherapy as a constituent [18, 19]. Therefore, it could naturally be inferred that when combined with fluorouracil or capecitabine, oxaliplatin might ameliorate long-term outcome of LARC as well.

The earliest randomized trial comparing chemotherapy regimens with or without oxaliplatin in LARC was the STAR-01 trial, in which combination of oxaliplatin and fluorouracil failed to improve tumor response [6]. In the following ACCORD 12 and NSABP R-04 trials, no improvement of 3- or 5- year DFS or OS was achieved through adding oxaliplatin to capecitabine or fluorouracil, either [7, 8]. The CAO/ARO/AIO-04 trial was the first study to support addition of oxaliplatin to fluorouracil and leucovorin (FOLFOX). This regimen increased 3-year DFS from 71.2 to 75.9% [9]. The FOWARC trial reported that FOLFOX regimen could also elevate pCR (14.0 to 27.5%) and downstaging (37.1 to 56.4%) rates of patients [20]. Similarly, the ADORE trial and a trial by Jiao et al. showed superiority of FOLFOX regimen in reducing DM and ameliorating DFS at 3 years [21, 22]. Meta-analyses provided further evidences for application of oxaliplatin. De Felice et al., found oxaliplatin to decrease risk of DM (HR = 0.76), through analysis of 4 trials [23]. And Zheng et al. analyzed data of 8 trails to report that oxaliplatin could brought a better DFS [24].

It was noteworthy that the COD in the STAR-01, ACCORD 12 and NSABP R-04 trials (360, 250 and 250 mg/m^2^) were much less than that in the CAO/ARO/AIO-04, FOWARC, ADORE trials and the trial by Jiao et al. (1000, 1020, 680, and 750–920 mg/m^2^) [6–9, 20–22]. Considering that TCC ≥ 8 was an independent predictor of better prognosis in our previous works [11], we speculated that enough COD might be necessary to attain ideal DM control and survival improvement, especially for patients receiving insufficient TCC (≤ 7). Hence, survival analyses stratified by TCC was performed in this study, to test our hypothesis. As expected, COD of 460–720 mg/m^2^ appeared as an independent predictor of better OS, MFS and DFS in the TCC ≤ 7 group. But in the TCC ≥ 8 group, COD of 720–780 mg/m^2^ failed to bring any benefit of OS, RFS, MFS or DFS. Currently, a considerable number of patients do not receive ACT, because of its uncertain necessity in LARC treated with NACRT [25, 26]. For these patients and those refusing or intolerable for ACT, NACT with a combination regimen containing oxaliplatin of ≥460 mg/m^2^ might be needed. Our study provided findings illuminating to conduct individualized treatment and clinical trials.

Raised risk of toxicities from oxaliplatin, especially severe PN, is another concern. A trial by Schmoll et al. showed that compared with fluorouracil plus leucovorin, CAPEOX regimen brought more grade 3/4 acute PN (11% vs. < 1%) without increasing other severe toxicities [27]. In the NSABP C-07 trial, the most common grade 3/4 acute toxicities were diarrhea, nausea and vomiting, whose incidences was not affected by oxaliplatin. FOLFOX regimen merely caused a higher incidence of grade 2+ PN (30.4% vs. 3.6%) [17]. Oppositely, in the STAR-01 trial, oxaliplatin resulted in more grade 3/4 acute diarrhea (15% vs. 4%) and asthenia (3% vs. 0%) but not grade 3/4 PN [6]. Although having a high COD, the CAO/ARO/AIO-04 trial reported that patients receiving regimens with and without oxaliplatin underwent similar acute toxicities, including PN [9]. In our study, 256 out of the 388 patients (66.0%) suffered from acute toxicities, among which 20.3% were of grade 3/4. PN was seen in 32.2% of the patients and all of grade 1/2. Comparison of toxicities revealed that in the TCC ≤ 7 group, COD ≥460 mg/m^2^ was associated with a higher incidence of overall acute toxicities, rather than PN or grade 3/4 toxicities. Interestingly, COD of ≥720 mg/m^2^ did not lead to more chemoradiotherapy-related toxicities in the TCC ≥ 8 group. Our results was in accordance with those of the CAO/ARO/AIO-04 trial. In other words, oxaliplaitn could be safe when COD was ≤780 mg/m^2^.

Indeed, there were 2 major limitations in this study. First, the retrospective nature of this study might bring some biases, like selection biases. But comparison of baseline characteristics showed that the patients with different CODs were comparable. And multivariate survival analysis could control biases to some extent. Second, its sample size was not large enough, for NACRT became mainstream treatment for LARC just from the year of 2012, even in the developed countries [28]. So we proposed that our results be further validated by prospective randomized controlled trials before popularization.

## Conclusions

For LARC treated with TCC ≤ 7, oxaliplatin of ≥460 mg/m^2^ might be needed to improve survival, though it might resulted in more mild acute toxicities. This finding is instructive though further validation is recommended.

## Supplementary information

**Additional file 1: Table S1.** Multivariate survival analysis in patients treated with total chemotherapy cycle ≥7.

## Data Availability

The datasets used and/or analyzed during the current study are available from the corresponding authors on reasonable request.

## Abbreviations

LARC: Locally advanced rectal cancer
NACRT: Neoadjuvant chemoradiotherapy
ACT: Adjuvant chemotherapy
DM: Distant metastasis
CAPEOX: Capecitabine plus oxaliplatin
pCR: pathological complete response
DFS: Disease-free survival
COD: Cumulative oxaliplatin dose
PN: Peripheral neuropathy
OS: Overall survival
MFS: Metastasis-free survival
TCC: Total chemotherapy cycle
NACT: Neoadjuvant chemotherapy
CT: Computed tomography
MRI: Magnetic resonance imaging
CEA: Carcinoembryonic antigen
CA19–9: Carbohydrate antigen 19–9
CTCAE: Common Terminology Criteria for Adverse Events
TME: Total mesorectal excision
TRG: Tumor regression grade
RFS: Recurrence-free survival
LR: Local recurrence
HR: Hazard ratios
CI: Confidence interval
FOLFOX: Oxalipaltin, fluorouracil plus leucovorin

## References

[1] Chen W, Sun K, Zheng R (2018). Cancer incidence and mortality in China, 2014. Chin J Cancer Res.

[2] Pang Y, Kartsonaki C, Guo Y (2018). Diabetes, plasma glucose and incidence of colorectal cancer in Chinese adults: a prospective study of 0.5 million people. J Epidemiol Community Health.

[3] Smith CA, Kachnic LA (2018). Evolving treatment paradigm in the treatment of locally advanced rectal Cancer. J Natl Compr Cancer Netw.

[4] Fokas E, Ströbel P, Fietkau R (2017). German Rectal Cancer Study Group. Tumor Regression Grading After Preoperative Chemoradiotherapy as a Prognostic Factor and Individual-Level Surrogate for Disease-Free Survival in Rectal Cancer. J Natl Cancer Inst.

[5] Roselló S, Papaccio F, Roda D (2018). The role of chemotherapy in localized and locally advanced rectal cancer: a systematic revision. Cancer Treat Rev.

[6] Aschele C, Cionini L, Lonardi S (2011). Primary tumor response to preoperative chemoradiation with or without oxaliplatin in locally advanced rectal cancer: pathologic results of the STAR-01 randomized phase III trial. J Clin Oncol.

[7] Gérard JP, Azria D, Gourgou-Bourgade S (2012). Clinical outcome of the ACCORD 12/0405 PRODIGE 2 randomized trial in rectal cancer. J Clin Oncol.

[8] Allegra CJ, Yothers G, O'Connell MJ (2015). Neoadjuvant 5-FU or Capecitabine Plus Radiation With or Without Oxaliplatin in Rectal Cancer Patients: A Phase III Randomized Clinical Trial. J Natl Cancer Inst.

[9] Rödel C, Graeven U, Fietkau R (2015). German rectal Cancer study group. Oxaliplatin added to fluorouracil-based preoperative chemoradiotherapy and postoperative chemotherapy of locally advanced rectal cancer (the German CAO/ARO/AIO-04 study): final results of the multicentre, open-label, randomised, phase 3 trial. Lancet Oncol..

[10] Sereno M, Gutiérrez-Gutiérrez G, Rubio JM (2017). Genetic polymorphisms of SCN9A are associated with oxaliplatin-induced neuropathy. BMC Cancer.

[11] Chang H, Yu X, Chen K (2018). Prognostic value of the cycle number of perioperative chemotherapy in Locoregionally advanced rectal Cancer: a propensity score matching analysis. J Cancer.

[12] Herzog T, Belyaev O, Chromik AM (2010). TME quality in rectal cancer surgery. Eur J Med Res.

[13] Trakarnsanga A, Gönen M, Shia J (2014). Comparison of tumor regression grade systems for locally advanced rectal cancer after multimodality treatment. J Natl Cancer Inst.

[14] World Health Organization (2011). The global prevalence of Anaemia in 2011. Geneva Switzerland WHO.

[15] McCarthy K, Pearson K, Fulton R, Hewitt J (2012). Pre-operative chemoradiation for non-metastatic locally advanced rectal cancer. Cochrane Database Syst Rev.

[16] Zou XC, Wang QW, Zhang JM (2017). Comparison of 5-FU-based and Capecitabine-based Neoadjuvant Chemoradiotherapy in patients with rectal Cancer: a meta-analysis. Clin Colorectal Cancer.

[17] Kjellström J, Kjellén E, Johnsson A (2005). In vitro radiosensitization by oxaliplatin and 5-fluorouracil in a human colon cancer cell line. Acta Oncol.

[18] Yothers G, O'Connell MJ, Allegra CJ (2011). Oxaliplatin as adjuvant therapy for colon cancer: updated results of NSABP C-07 trial, including survival and subset analyses. J Clin Oncol.

[19] André T, de Gramont A, Vernerey D (2015). Adjuvant fluorouracil, Leucovorin, and Oxaliplatin in stage II to III Colon Cancer: updated 10-year survival and outcomes according to BRAF mutation and mismatch repair status of the MOSAIC study. J Clin Oncol.

[20] Deng Y, Chi P, Lan P (2016). Modified FOLFOX6 with or without radiation versus fluorouracil and Leucovorin with radiation in Neoadjuvant treatment of locally advanced rectal Cancer: initial results of the Chinese FOWARC multicenter, open-label, randomized three-arm phase III trial. J Clin Oncol.

[21] Hong YS, Nam BH, Kim KP (2014). Oxaliplatin, fluorouracil, and leucovorin versus fluorouracil and leucovorin as adjuvant chemotherapy for locally advanced rectal cancer after preoperative chemoradiotherapy (ADORE): an open-label, multicentre, phase 2, randomised controlled trial. Lancet Oncol.

[22] Jiao D, Zhang R, Gong Z (2015). Fluorouracil-based preoperative chemoradiotherapy with or without oxaliplatin for stage II/III rectal cancer: a 3-year follow-up study. Chin J Cancer Res.

[23] De Felice F, Benevento I, Magnante AL (2017). Clinical benefit of adding oxaliplatin to standard neoadjuvant chemoradiotherapy in locally advanced rectal cancer: a meta-analysis: Oxaliplatin in neoadjuvant treatment for rectal cancer. BMC Cancer.

[24] Zheng J, Feng X, Hu W (2017). Systematic review and meta-analysis of preoperative chemoradiotherapy with or without oxaliplatin in locally advanced rectal cancer. Medicine (Baltimore).

[25] Breugom AJ, Swets M, Bosset JF (2015). Adjuvant chemotherapy after preoperative (chemo)radiotherapy and surgery for patients with rectal cancer: a systematic review and meta-analysis of individual patient data. Lancet Oncol..

[26] Dossa F, Acuna SA, Rickles AS (2018). Association between adjuvant chemotherapy and overall survival in patients with rectal Cancer and pathological complete response after Neoadjuvant chemotherapy and resection. JAMA Oncol.

[27] Schmoll HJ, Cartwright T, Tabernero J (2007). Phase III trial of capecitabine plus oxaliplatin as adjuvant therapy for stage III colon cancer: a planned safety analysis in 1,864 patients. J Clin Oncol.

[28] Sineshaw HM, Jemal A, Thomas CR (2016). Changes in treatment patterns for patients with locally advanced rectal cancer in the United States over the past decade: an analysis from the National Cancer Data Base. Cancer..

