# Supplementary material for: Optimize the dose of oxaliplatin for locally advanced rectal cancer treated with neoadjuvant chemoradiotherapy followed by radical surgery and adjuvant chemotherapy
Source: BMC Cancer. 2020 Jun 1;20:498. doi: 10.1186/s12885-020-06988-x (PMC7268650; doi:10.1186/s12885-020-06988-x)
Supplement: Supplementary file 1 — Additional file 1: Table S1. Multivariate survival analysis in patients treated with total chemotherapy cycle ≥7. [file 12885_2020_6988_MOESM1_ESM.docx]

**Table S1 Multivariate survival analysis in patients treated with total chemotherapy cycle ≥ 7.**

| Factors of OS | *P* value | HR | 95% CI |
| --- | --- | --- | --- |
| Pathological stage (ypIII-II vs. ypI-0) | 0.264 | 2.105 | 0.570-7.813 |
| TRG (5-3 vs. 2-1) | 0.777 | 1.208 | 0.327-4.464 |
| Factors of MFS | ***P* value** | **HR** | **95% CI** |
| Pathological stage (ypIII-II vs. ypI-0) | 0.185 | 2.132 | 0.696-6.536 |
| TRG (5-3 vs. 2-1) | 0.712 | 1.235 | 0.403-3.774 |
| Factors of DFS | ***P* value** | **HR** | **95% CI** |
| Pathological stage (ypIII-II vs. ypI-0) | 0.181 | 2.110 | 0.707-6.289 |
| TRG (5-3 vs. 2-1) | 0.757 | 1.189 | 0.398-3.546 |

Abbreviations: OS, overall survival; MFS, metastasis-free survival; DFS, disease-free survival; HR, hazard ratio; CI, confidence interval; TRG, tumor regression grade.
